# 

**Published:** 1899-03

**Authors:** 


### Austin District Medical Society.

The forty-sixth quarterly meeting of the Austin District Medical Society will be held in the Knights of Pythias hall, over 106 East Seventh Street, Austin, Texas, Thursday, March 23, 1899.

OFFICERS.

J. C. Anderson, M. D., President, Granger.
H. B. Hill, M. D., First Vice-President, Austin.
J. S. Watson, M. D., Second Vice-President, Manor.
S. E. Hudson, M. D., Secretary and Treasurer, Austin.

You are invited to be present and participate in the following

PROGRAM.

1. "Etiology and Treatment of Endometritis," by Dr. J. S. Watson; discussion opened by Drs. F. A. Maxwell and W. J. Mathews.
2. "Tonsillitis, Acute and Chronic," by Dr. A. Nowlin; discussion opened by Drs. H. B. Hill and J. S. Poyner.
3. "Entero Collitis of Children," by Dr. W. E. Holtzclaw; discussion opened by Drs. J. M. Strayhorn and R. M. Wickline.
4. "Cerebro-Spinal Meningitis," By Dr. Matthew M. Smith; discussion opened by Drs. B. M. Worsham and Thos. D. Wooten.
5. "Unusual Complications in a Case of Typhoid Fever," by Dr. H. B. Granberry; discussion opened by Drs. W. T. Richmond and Jno. R. Hunter.

J. C. ANDERSON, President.

S. E. HUDSON, Secretary.

